# Reliability of [^18^F]FDG PET/CT for post-treatment surveillance of non-small cell lung cancer: agreement among multiple centers

**DOI:** 10.1007/s00259-025-07420-x

**Published:** 2025-06-21

**Authors:** Kasper Foged Guldbrandsen, Markus Nowak Lonsdale, Hanne Marie Nellemann, Catharina Mølgaard Bylov, Joan Fledelius, Karin Hjorthaug, Barbara Jolanta Jørgensen, Martin Krakauer, Mette Schødt, Peter Michael Gørtz, Mie Kiszka Nielsen, Anne Lerberg Nielsen, Annemarie Gjelstrup Amtoft, Elisabeth Albrecht-Beste, Danijela Dejanovic, Marie Josée Zareh Lausten-Thomsen, Paw Christian Holdgaard, Magdalene Kubik, Søren Steen Nielsen, Oke Gerke, Torben Riis Rasmussen, Barbara Malene Fischer

**Affiliations:** 1 Department of Clinical Physiology and Nuclear Medicine, Copenhagen University Hospital - Rigshospitalet, Copenhagen, Denmark; 2 Department of Clinical Physiology and Nuclear Medicine, Copenhagen University Hospital - Bispebjerg and Frederiksberg, Copenhagen, Denmark; 3 Department of Radiology, Aarhus University Hospital, Aarhus, Denmark; 4 Department of Nuclear Medicine & PET-Centre, Aarhus University Hospital, Aarhus, Denmark; 5 Department of Radiology, Copenhagen University Hospital - Bispebjerg and Frederiksberg, Copenhagen, Denmark; 6 Department of Radiology, Copenhagen University Hospital - Herlev and Gentofte, Hellerup, Denmark; 7 Department of Nuclear Medicine, Copenhagen University Hospital - Herlev and Gentofte, Hellerup, Denmark; 8 Department of Radiology, Odense University Hospital, Odense, Denmark; 9 Department of Nuclear Medicine, Odense University Hospital, Odense, Denmark; 10 Department of Clinical Medicine, Faculty of Health and Medical Sciences, University of Copenhagen, Copenhagen, Denmark; 11 Department of Radiology, University Hospital of Southern Denmark, Lillebaelt Hospital - Vejle, Vejle, Denmark; 12 Department of Nuclear Medicine, University Hospital of Southern Denmark, Lillebaelt Hospital - Vejle, Vejle, Denmark; 13 Department of Nuclear Medicine, Aalborg University Hospital, Aalborg, Denmark; 14 Research Unit of Clinical Physiology and Nuclear Medicine, Department of Clinical Research, University of Southern Denmark, Odense, Denmark; 15 Department of Respiratory Medicine and Allergy, Aarhus University Hospital, Aarhus, Denmark; 16 Department of Clinical Medicine, Aarhus University, Aarhus, Denmark

**Keywords:** [^18^F]FDG PET/CT, Lung cancer, Surveillance, Recurrence, Interobserver agreement

## Abstract

**Purpose:**

Fluorine-18 fluorodeoxyglucose positron emission tomography/computed tomography ([^18^F]FDG PET/CT) has shown promise for post-treatment surveillance in patients with non-small cell lung cancer (NSCLC). This study evaluated interobserver agreement of PET/CT interpretation for NSCLC surveillance in a multicenter setting.

**Methods:**

Nine teams from seven centers, each team consisting of a nuclear medicine specialist and a radiologist, participated in the study. A total of 150 PET/CT scans were selected, and each was independently reviewed by two randomly assigned teams. Scans were performed six months post-treatment for scheduled recurrence assessment in stage Ia-IIIc NSCLC patients. Each scan was evaluated for suspicion of recurrence using two methods; without any pre-specified criteria (conventional assessment) and using pre-specified, qualitative criteria (Hopkins criteria). Both scoring methods were compared to a reference standard to assess accuracy.

**Results:**

Conventional assessment showed moderate interobserver agreement (κ = 0.55, 95% CI 0.41–0.69; 79% overall agreement) for the diagnosis of recurrence. Hopkins criteria demonstrated substantial agreement (κ = 0.61, 95% CI 0.45–0.77; 87% overall agreement). There was no difference in the area under the curve (AUC) between conventional assessment (0.80, 95% CI 0.72–0.88) and Hopkins criteria (0.82, 95% CI 0.74–0.90) compared to the reference standard (*p* = 0.21).

**Conclusions:**

Interobserver agreement for [^18^F]FDG PET/CT interpretation in NSCLC surveillance was moderate to substantial. While applying pre-specified reporting criteria did not significantly improve the agreement, it did not hinder the diagnostic accuracy. Efforts to reduce the variability of reporting, including continuous training and structured reporting, could improve the clinical impact of this technology.

## Introduction

Patients with non-small cell lung cancer (NSCLC) face a significant risk of recurrence after curative treatment, with rates ranging from 20 to 50% depending on the initial disease stage and treatment modality [1]. Early detection of recurrence may enable better treatment options and potentially improve survival [2]. Consequently, post-treatment surveillance is recommended by most guidelines, although the optimal surveillance strategy remains a subject of debate [3].

While most guidelines recommend scheduled surveillance with computed tomography (CT), these recommendations are primarily based on expert opinion due to the lack of high-quality evidence supporting one surveillance approach over another [4]. In recent years, fluorine-18 fluorodeoxyglucose positron emission tomography/computed tomography ([18 F]FDG PET/CT) has been explored as a potential alternative to CT for post-treatment surveillance in NSCLC [5].

Early studies on [^18^F]FDG PET/CT in NSCLC surveillance reported encouraging results. A meta-analysis of retrospective trials demonstrated superior diagnostic performance for detecting recurrence compared to CT [6]. Studies have reported a near-perfect diagnostic accuracy, with sensitivity and specificity rates of 99% and 98%, respectively [7]. Additionally, [^18^F]FDG PET/CT has shown promise in differentiating treatment-related changes from recurrence, which is often challenging, especially after radiotherapy [8].

However, recent prospective trials have produced less favorable results. A randomized controlled trial involving 96 patients found no significant difference between [^18^F]FDG PET/CT and CT in terms of recurrence detection or the proportion of patients receiving curative treatment for recurrence [9]. These findings were corroborated by a larger randomized trial of 750 patients, which showed no benefit for [^18^F]FDG PET/CT in recurrence detection, rate of curative treatment, or overall survival when compared to CT [10].

The discrepancy between early retrospective studies and recent prospective trials underscores the need to examine factors that could limit the performance of this technology when applied in broader clinical settings. An often overlooked aspect is the variability in image interpretation among readers, which can limit the effectiveness of a diagnostic tool [11]. The purpose of this study was to evaluate the interrater reliability of [^18^F]FDG PET/CT interpretation for NSCLC recurrence detection in a multicenter setting with multiple observers, using prospectively collected imaging data from the SUPE_R trial [10]. Additionally, we aimed to assess how pre-specified criteria for recurrence assessment might affect reliability and accuracy.

## Materials and methods

### Population and observers

This interobserver variability study utilized [^18^F]FDG PET/CT scans from patients with stage IA-IIIC NSCLC who had completed curative treatment. The scans were obtained approximately six months after treatment as part of scheduled surveillance, with no prior suspicion of recurrence. The patients were part of the PET/CT arm of the SUPE_R trial (ClinicalTrials.gov NCT03740126). Full details of the SUPE_R trial and primary results have been published previously [10].

Sample size was determined using the confidence interval approach of Donner and Rotondi [12], using the R package kappaSize [13]. Based on an expected κ of 0.73 [14] an acceptable lower confidence limit of 0.50, and an expected marginal distribution of 80% benign scans, 10% equivocal, and 10% suspicious for recurrence, with α = 0.05 and power of 80%, a minimum of 147 subjects were required. One hundred fifty scans from 150 patients were randomly selected, with one scan per patient obtained at the 6-month time point. The selection was stratified by recurrence suspicion based on the original report, ensuring that approximately 15% of the selected scans had findings suspicious for recurrence, matching the expected rate in the population. The 6-month surveillance time point was chosen as the risk of recurrence is highest during the first 12 months after curative treatment, with the hazard rate peaking at approximately 6 months [15].

Nine teams from seven nuclear medicine departments across Denmark participated in the study. Each team consisted of a board-certified nuclear medicine physician and a board-certified radiologist, both with extensive experience in PET imaging and lung cancer evaluation. Two teams were randomly assigned to independently review each scan, with the constraint that no two teams from the same department reviewed the same scan and no team rated scans originating from their own department. This was done to ensure independent review and avoid recall bias.

The scans were centrally anonymized and assigned unique study IDs. For each scan, the corresponding baseline scan (obtained for staging prior to therapy) was also anonymized and made available for comparison. No other imaging was provided. The anonymized scans were distributed to the selected departments and loaded into their respective Picture Archiving and Communication Systems (PACS) used for routine clinical practice.

### Scoring methods

Teams reviewed their assigned scans according to their usual practice, individually or side by side. The final rating for each team was based on a consensus reading between the two team members. Ratings were recorded in a REDCap database [16, 17], where selected patient characteristics, including stage, treatment, histology, age, and gender, were presented to the reviewers.

Each scan was scored in three steps. First, teams evaluated all abnormal findings by anatomical site, scoring them for both FDG uptake and suspicion of recurrence. FDG-uptake was classified on a 4-point scale (normal, mildly increased, moderately increased, markedly increased), with teams free to use quantitative and qualitative assessment methods. Suspicion of recurrence was rated on a 5-point scale: benign, most likely benign, either benign or recurrence, most likely recurrence or definite recurrence.

Next, teams assessed the overall suspicion of recurrence for each scan on a 3-point scale, considering both PET and CT findings equally. The three categories were: no suspicion of recurrence, equivocal for recurrence, or suspicious for recurrence. For this method, referred to as the “conventional assessment”, teams were instructed to apply their usual clinical criteria for diagnosing recurrence without pre-defined study-specific guidelines.

Finally, teams applied the Hopkins criteria for evaluation of lung cancer recurrence [18]. This approach involved a qualitative evaluation of FDG uptake in abnormal findings across three regions: primary tumor site, regional mediastinal nodes, and distant sites (including contralateral lung). For each region, teams assessed the FDG uptake using a 5-point scale, visually comparing the activity to the mediastinal blood pool and liver uptake (Table 1). Teams were instructed to disregard incidental findings likely unrelated to lung cancer. The final recurrence assessment was based on the highest score among all three regions, with scores of 4 or 5 considered positive for recurrence.

**Table 1:** Hopkins criteria. Each anatomical site (primary tumor, mediastinum and metastasis) is scored on a 5-point scale, and the final score is positive if any site score is above 3. FDG = Fluorine-18 Fluorodeoxyglucose

Score	Description
1	FDG uptake ≤ mediastinal blood pool
2	Focal FDG uptake > mediastinal blood pool but ≤ liver
3	Diffuse FDG uptake > mediastinal blood pool or liver
4	Focal FDG uptake higher than liver
5	Focal FDG uptake markedly higher than liver

To compare the relative accuracy of each rating method, we evaluated the results against a common reference standard. This standard was defined as the presence or absence of recurrence diagnosed within six months after the evaluated scan, as recorded in the SUPE_R study. Recurrence diagnoses were based on histological confirmation when possible or determined through multidisciplinary team assessment and imaging follow-up.

### Image acquisition

PET/CT scans were performed using standardized protocols drawn up at each department, adhering to European Association of Nuclear Medicine (EANM) recommendations [19]. Patients fasted for at least 4 h prior to the examination, followed by an [^18^F]FDG injection of 3–4 MBq/kg. Images were acquired 60 min post-injection, with scans covering the area from vertex to mid-thigh. Contrast-enhanced CT was used when not contraindicated.

#### Statistical analysis

The primary endpoint was overall agreement and Fleiss’ kappa (κ) for recurrence suspicion using the 3-point conventional assessment scale. Secondary endpoints included agreement using Hopkins criteria, agreement by anatomical site, and comparison of the accuracy of each rating method against the reference standard.

Interobserver agreement was assessed using overall agreement and Fleiss’ weighted kappa with linear weights [20, 21]. Fleiss’ kappa was chosen as it allows for a non-fully crossed study design with non-unique raters, giving the possibility of including a larger sample of scans compared to a fully crossed design, given the same total number of ratings. The agreement was categorized according to Landis and Koch (1977): 0.00–0.20 slight, 0.21–0.40 fair, 0.41–0.60 moderate, 0.61–0.80 substantial, and 0.81–1.00 almost perfect agreement [22]. Overall agreement was defined as the proportion of scans with identical ratings. Receiver Operating Characteristic (ROC) analysis was performed to evaluate the accuracy of each scoring method compared to the reference standard.

All statistical analyses were conducted using R software (version 4.4.1), with the irrCAC package for estimation of Fleiss’ kappa [23]. Two-sided p-values were used, with statistical significance set at *p* < 0.05.

## Results

### Study population

Out of 150 patients, 85 (57%) were female, with a mean age of 68.8 years (SD 8.6; Table 2). Adenocarcinoma was the predominant histological type, accounting for 110 patients (73% of the 150 patients). Stage distribution, according to the 8th edition of the American Joint Committee on Cancer (AJCC) TNM staging system, comprised 109 patients (73%) with stage I, 21 (14%) with stage II, and 20 (13%) with stage III disease. Initial curative treatment for NSCLC included surgery (118 patients, 79%), stereotactic body radiation therapy (SBRT; 21 patients, 14%), and chemoradiotherapy (11 patients, 7%). The original reports indicated suspicion of recurrence in 23 patients (15%), with recurrence confirmed within six months in 18 patients (12%).

**Table 2:** Baseline characteristics of included patients. Reported as counts and percentage of included patients unless otherwise specified. SD = Standard deviation; pet/ct = positron emission tomography/computed tomography

Characteristic	All patients
N	150
Age - mean (SD)	68.8 (8.6)
Sex	
Female	85 (57%)
Male	65 (43%)
Histology	
Adenocarcinoma	110 (73%)
Squamous cell carcinoma	35 (23%)
Other	5 (3%)
Stage	
I	109 (73%)
II	21 (14%)
III	20 (13%)
Treatment	
Surgery	118 (79%)
Stereotactic Body Radiation Therapy	21 (14%)
Chemoradiotherapy	11 (7%)
6-month surveillance PET/CT	
Recurrence suspected (original report)	23 (15%)
Recurrence diagnosed within six months	18 (12%)

### Interobserver agreement

All 150 scans were successfully evaluated, with each scan independently reviewed by two teams randomly selected from the pool of nine teams. Tables 3 and 4 show the distribution of ratings for 3-point conventional assessment scale and the 5-point Hopkins criteria scale, respectively. For the conventional assessment, there was disagreement between teams in 31 of 150 scans, and Fleiss’ kappa indicated moderate agreement (κ = 0.55, 95% confidence interval [CI] 0.41–0.69; overall agreement 79%). Out of 31 scans with disagreement, 18 had findings only rated as suspicious for recurrence by one team, six had abnormal findings considered more suspicious by one team, and 7 had findings suspicious of malignancy but disagreement on whether the finding was related to lung cancer. By treatment, agreement was moderate post-surgery (κ = 0.46, 95% CI 0.27–0.64), substantial post-SBRT (κ = 0.73, 95% CI 0.45–1), and substantial post-chemoradiotherapy (κ = 0.71, 95% CI 0.26–1.00). For Hopkins criteria, with a score of 4 or 5 considered positive, teams disagreed in 19 of 150 scans, and Fleiss’ kappa indicated substantial agreement (κ = 0.61, 95% CI 0.45–0.77; overall agreement 87%).

Analysis of agreement by team revealed some variability in interobserver agreement across different teams (κ ranging from 0.31 to 0.83 for conventional assessment; Table 5), though no outliers could be identified.

**Table 3:** Distribution of ratings using conventional assessment (3-point scale). Each scan was independently reviewed by two teams, randomly selected from a pool of nine teams

	2nd rating		
1 st rating	No recurrence	Equivocal	Recurrence
No recurrence	100	8	15
Equivocal		3	8
Recurrence			16

**Table 4:** Distribution of ratings using Hopkins criteria (5-point scale). Each scan was independently reviewed by two teams, randomly selected from a pool of nine teams

	2nd rating				
1 st rating	1	2	3	4	5
1	75	9	9	6	4
2		4	9	4	0
3			4	3	2
4				4	3
5					14

**Table 5:** Interobserver agreement for recurrence suspicion by assessment team and scoring method

			Conventional Assessment		Hopkins Criteria	
Site	Team	Number of Scans	Overall Agreement (%)	Fleiss’ kappa (95% CI)	Overall Agreement (%)	Fleiss’ kappa (95% CI)
Site A	Team 1	33	70%	0.43 (0.12–0.74)	85%	0.57 (0.21–0.92)
Site B	Team 2	33	73%	0.57 (0.31–0.82)	82%	0.54 (0.20–0.88)
Site B	Team 3	34	94%	0.83 (0.59–1.00)	91%	0.67 (0.31–1.00)
Site C	Team 4	33	70%	0.41 (0.07–0.75)	85%	0.63 (0.32–0.94)
Site D	Team 5	33	85%	0.59 (0.24–0.94)	88%	0.59 (0.21–0.97)
Site E	Team 6	34	88%	0.77 (0.54–1.00)	97%	0.87 (0.61–1.00)
Site F	Team 7	34	79%	0.59 (0.31–0.87)	85%	0.61 (0.28–0.94)
Site F	Team 8	33	85%	0.53 (0.14–0.91)	88%	0.43 (0.06–0.92)
Site G	Team 9	33	70%	0.31 (0.06–0.69)	85%	0.52 (0.13–0.91)
**Total**	**All teams**	**150**	**79%**	**0.55 (0.41–0.69)**	**87%**	**0.61 (0.45–0.77)**

### Agreement by anatomical site

Abnormal findings were identified at the primary tumor site (*n* = 22), remaining lungs (*n* = 35), mediastinum (*n* = 26), and other metastatic sites (*n* = 12; Table 6). The agreement was moderate for the primary tumor site (κ = 0.58, 95% CI 0.42–0.74), remaining lungs (κ = 0.44, 95% CI 0.24–0.64), and the mediastinum (κ = 0.60, 95% CI 0.40–0.80).

**Table 6:** Interobserver agreement for suspicion of recurrence by anatomical site. Category “other metastatic sites” include brain, adrenals, liver and miscellaneous findings

Site	Suspected recurrence (*n*)	Overall agreement	Fleiss’ kappa (95% CI)
Primary tumor site	22	84%	0.58 (0.42–0.74)
Lung (excl. primary tumor)	35	85%	0.44 (0.24–0.64)
Pleura	3	99%	0.8 (0.35–1)
Mediastinum	26	90%	0.6 (0.4–0.8)
Bones	4	98%	0.39 (0.1–0.88)
Other metastatic sites	12	99%	0.48 (0.14–0.83)

### ROC analysis

**Fig. 1:**
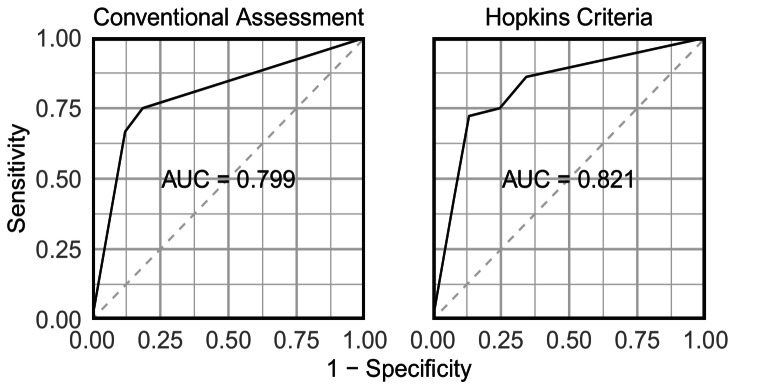
ROC curves for conventional assessment (3-point scale) and Hopkins criteria (5-point scale). There was no significant difference in AUC (*p* = 0.21)

The area under the curve (AUC) of each rating compared to the reference standard was 0.80 (95% CI 0.72–0.88) for conventional assessment and 0.82 (95% CI 0.74–0.90) for Hopkins criteria (Fig. 1). The difference in AUC between the conventional assessment and Hopkins criteria was not statistically significant (*p* = 0.21). Compared to the AUC of original reports (0.85; 95% CI 0.77–0.93), neither the conventional assessment (*p* = 0.94) nor the Hopkins criteria (*p* = 0.81) showed a statistically significant difference.

## Discussion

### Main findings

In this multicenter study evaluating interobserver agreement of [^18^F]FDG PET/CT interpretation for NSCLC recurrence detection, we found moderate agreement using conventional assessment (κ = 0.55, 95% CI 0.41–0.69) and substantial agreement using pre-specified Hopkins criteria (κ = 0.61, 95% CI 0.45–0.77). The diagnostic accuracy was comparable between both methods (AUC 0.80 vs. 0.82, *p* = 0.21).

### Comparison with prior studies

To our knowledge, only one study has previously evaluated interobserver variability of PET/CT reporting without pre-specified criteria for NSCLC recurrence detection, finding almost perfect agreement (κ = 0.89) [24], which is higher than what we found using conventional assessment (κ = 0.55). Similarly, our results using Hopkins criteria (κ = 0.61) showed lower agreement than previous studies that reported kappa values ranging from 0.73 to 0.89 [14, 18] using the same criteria. In our study, observers applied the Hopkins criteria immediately after the conventional assessment, potentially influencing the second interpretation, increasing the similarity in results between the two assessment methods. However, the generally lower agreement in our study compared to previous reports is also likely due to differences in study design and setting. Previous studies reported the agreement between two observers at a single institution, which could be expected to be higher than the agreement between raters from multiple institutions.

A few studies have evaluated the interobserver agreement for [^18^F]FDG PET/CT in NSCLC staging. In a study of 100 patients, a high agreement was reported for both N staging (κ = 0.75–0.81) and M staging (κ = 0.90–0.93) [25]. Other studies have reported moderate (κ = 0.65) to almost perfect agreement (κ = 0.92–0.96) for mediastinal lymph node evaluation [26, 27]. While lung cancer staging relies heavily on well-established criteria for reporting, such as the TNM system, recurrence assessment relies more on pattern recognition and clinical judgment, which could increase the variability in reporting.

We did not examine interobserver agreement for CT in our study, but studies on follow-up CT after SBRT have shown conflicting results, reporting fair (κ = 0.20–0.30) to almost perfect agreement (κ > 0.85) among observers [28, 29]. Both PET/CT and CT imaging can be particularly challenging to interpret after radiotherapy due to treatment-related changes [30, 31]. Recent joint EANM/SNMMI/ESTRO guidelines emphasize the importance of standardized interpretation and multidisciplinary collaboration to address the challenges faced in lung cancer patients treated with radiation therapy [32]. Interestingly, we found substantial agreement post-SBRT (κ = 0.73) and post-chemoradiotherapy (κ = 0.71) compared to only moderate agreement post-surgery (κ = 0.46). A previous study of FDG PET/CT scans for response assessment in post-chemoradiotherapy patients also found good reliability, reporting substantial to almost perfect agreement (κ = 0.76–0.87) using various response assessment criteria [33]. The higher agreement in post-radiotherapy patients might be attributed to more overt changes in FDG uptake compared to the more subtle changes typically seen post-surgery, making interpretation more consistent among readers.

### Strength and limitations

The primary strength of our study is the multicenter design with multiple raters and a comparatively large patient population, which better captures the variability that emerges when an imaging method is applied across diverse clinical settings. Additionally, the use of prospectively collected data from the SUPE_R trial reduces some of the potential biases associated with purely retrospective analyses.

Our study also has some limitations. First, the lack of access to all previous imaging data and clinical information for observers, which often guide diagnosis, may have contributed to increased variability in reporting. However, this limitation did not impact the diagnostic accuracy of the scoring methods in comparison to the reference standard, which was comparable to that of the original reports (*p* = 0.94 for conventional assessment and *p* = 0.81 for Hopkins criteria). Second, while an effort was made to capture real-world variability of reporting, using a 3-point scoring system for assessing recurrence does not fully reflect the nuanced information provided in complete, original reports. Third, while we included patients with different stages and treatment modalities to increase the generalizability, this heterogeneity also limits the applicability of our results to specific patient subgroups.

### Clinical implications and future directions

The moderate interobserver agreement observed in our study does not provide conclusive evidence regarding the relative merits of [^18^F]FDG PET/CT compared to CT for post-treatment surveillance in NSCLC. However, it may help explain why earlier, promising results have been challenging to replicate when evaluated in prospective trials [9, 10]. These results highlight that efforts to reduce variability in reporting, such as through shared learning and training, might improve the clinical impact of this technology. While the Hopkins criteria did not significantly improve reliability in our study, the comparable accuracy between methods suggests that such structured approaches warrant further investigation as potential tools for standardizing interpretation. Finally, these findings underscore the importance of diagnostic harmonization through close collaboration between diagnostic and clinical specialties, as achieved through multidisciplinary team meetings, which is standard practice in Denmark for evaluating suspected recurrence and recommended internationally for quality lung cancer care [34].

## Data Availability

The datasets generated during and/or analyzed during the current study are available from the corresponding author on reasonable request.
